# Efficacy of *Lagenidium giganteum* (Couch) metabolites for control *Anopheles stephensi* (Liston) a malaria vector

**DOI:** 10.1186/1475-2875-9-S2-P46

**Published:** 2010-10-20

**Authors:** Gavendra Singh, Soam Prakash

**Affiliations:** 1 Environmental and Advanced Parasitology and Vector Control Biotechnology Laboratories, Department of Zoology, Dayalbagh Educational Institute, Dayalbagh, Agra-282 005, India

## Background

**Figure 1:**
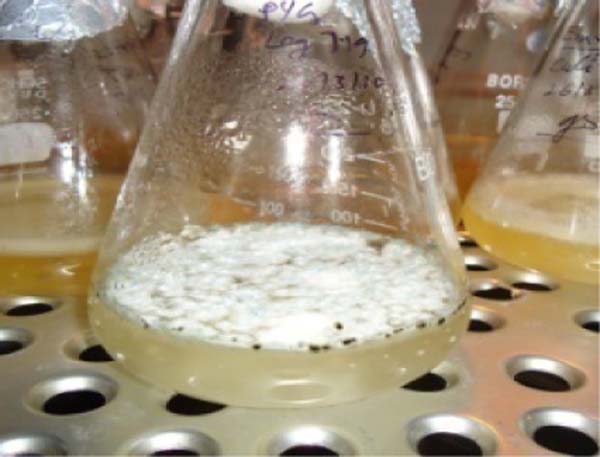
The culture of *L. giganteum* in Peptone yeast extract glucose (PYG) broth in the laboratory.

**Figure 2:**
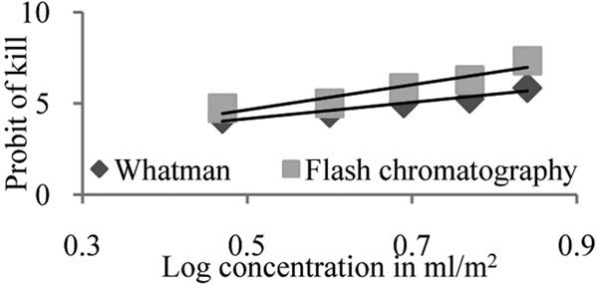
Probit regression line depicting relationship between probit of kill and log dose concentrations of metabolites of *L. giganteum* after 15 h exposure *for An. stephensi*.

**Figure 3:**
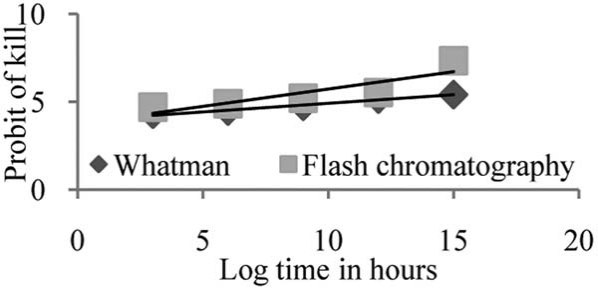
Probit regression line depicting relationship between probit of kill and log time of *L. giganteum* for *An. stephensi*.

*Lagenidium giganteum* (Couch) a water mold of class Oomy-cetes, is a facultative parasite of mosquito. The metabolites of L. *giganteum* were tested for control of *Anopheles stephensi.* This fungus was grown in Peptone yeast extract glucose (PYG) broth in the laboratory at 25±2°C, and relative humidity was maintained at 75±5% for 15±2 days. The filtration process of metabolites was done using Whatman-1 filter paper and then with Flash chromatography. These purified metabolites were spray at five (3,4,5,6,7 ml/m^2^) different statistically significant concentrations. The efficacy after whatman-1 the LC_50_-5ml/m^2^, LC_90_-7.07 ml/m^2^ and LC_99_-10 ml/m2 values were found effective after exposure of 1 5h. LT_50_-10.23 h and LT_90_-16.59 h were recorded. Similarly, efficacy after Flash chromatography the LC_50_-4 ml/m^2^, LC_90_-6 ml/m^2^ and LC_99_-6.76ml/m2 values were found effective after exposure of 15h. The LT_50_-6.16h and LT_90_-12.02h were observed. This study reveals the metabolites of *L.giganteum* could be used for control of *An. stephensi* a malaria vector. Figures 1, 2, 3

## Conclusion

Present study shows a high potential utility of fungal metabolites for complementing existing adult mosquito control measures. This approach demonstrates that the fugal metabolites have potential as a new strategy for vector control. It could be successful tool for controlling malaria vector in tropical countries with more community trials. Mosquitoes that are resistant to insecticides remain susceptible to fungal infection (Knols et al. 2010). Recently metabolites of *F.oxysporum* show significant pathogenicity against the larvae of *An. stephensi* in laboratory (Prakash et al. 2010). Regardless, the use of fungi *L. giganteum* to control population of *An. stephensi* mosquitoes clearly offers significant promise as a novel biologically based strategy to be integrated with other control measures to reduce global rate of malaria transmission. Also Scholte et al. (2005) have used fungal spores as adulticidal to control vector population in African villages, so fungal metabolites can be used as novel liquid adulticide.
